# An ultra performance liquid chromatography coupled with high resolution mass spectrometry method for the screening of cianotoxins content in drinking water samples

**DOI:** 10.1016/j.mex.2020.101001

**Published:** 2020-07-21

**Authors:** Giorgia di pofi, Gabriele Favero, Federica Nigro di Gregorio, Emanuele Ferretti, Luca lucentini

**Affiliations:** a Italian National Health Institute, Department of Environment and Health - Section of Water and Health, Viale Regina Elena, 299 – 00161 Rome, Italy; b University of Rome “Sapienza”, Department of Chemistry and Drug Technologies, Piazzale Aldo Moro, 5 00185, Rome, Italy

**Keywords:** Drinking water, Mass spectrometry, Microcystins

## Abstract

The incidence of cyanobacterial harmful algal blooms (CHABs) and their potentially toxic secondary metabolites is increasing in Italy and worldwide and several studies demonstrated that the climate change may be playing a role.

The method described in this work allows to detect simultaneously 21 cyanotoxins of different classes (including 12 Microcystins, 5 Microginins, 2 Cyanopeptolins, and 2 Anabaenopeptins) in water samples for human consumption by an Ultra Performance Liquid Chromatography coupled with a Q-TOF mass spectrometer.

Water samples were freezed, filtered, spiked with Nodularin used as internal standard (I.S.) and then extracted with a SPE Carboghaph 4 cartridge.

The extracted sample were analysed injecting 10 µL into the UPLC-HRMS/MS system: the chromatographic separation was obtained using acetonitrile and water as mobile phases, both containing 10 mM formic acid, and a UPLC C18 column, acquiring the experiments in positive ionization and Sensitivity Mode.

The described method allows to separate and detect all the selected analytes in short time of analysis (16 minutes) with a good resolution for all the analytes.

• simultaneous determination of 21 cyanotoxins belonging to different classes in water sample
• low injection volume
• short time of analysis

## Graphical abstract

**Figure:**
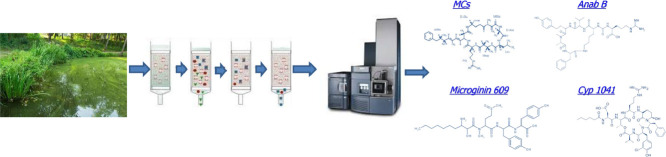

**Specifications Table**

**Table:** 

Subject Area:	Chemistry
More specific subject area:	*Analytical chemistry*
Method name:	*High resolution mass spectrometry*
Name and reference of original method:	
Resource availability:	*Acetonitrile, MilliQ, formic acid, cyanotoxins standard, Nodularin standard, Carbograph 4 cartridges, Dichloromethane, methanol, TFA, HCl, vacuum pump*

## Method details

This work describes an advanced analytical method for simultaneous determination of 21 cyanotoxins of four different class, including 12 Microcystins (MC), 2 Cyanopeptolins(CYP),2 Anabaenopeptins (ANAB) and 5 Microginins (MICRO).

For the analysis of total Cyanotoxins content, 250 mL of water sample were freezed, filtered, spiked with Nodularin (used as internal standard) at 1 µg/L and then extracted using Carbograph 4 SPE cartridge.

For the sample extraction a previous method [1] was updated adding Cyanopeptolins, Anabeanenopeptins and Microginins as analytes and the extraction efficiency was optimised for all the selected cyanotoxins.

All cyanotoxins standards were purchased by Alexis® Biochemicals (La Jolla, 96 CA, USA) while the reagents used were RS grade and the water was obtained from a Milli-Q system (Millipore Bedford, MA, USA).

The instruments used in this work were:

• Acquity UPLC system (Waters Corp., Milford, MA, USA)
• Acquity UPLC BEH C18 column (2,1 mm ID × 100 mm, 1,7 µm, Waters Corp., Milford, MA, USA)
• XEVO G2S Q-TOF (Waters Corp., Milford, MA, USA)

The presence and the quantization of the selected analytes were obtained used:

• a standard solution containing 0.1 μg/L of all the analytes and 1 μg/L of Nodularin used as internal standard (IS), prepared in 70:30 H_2_O: ACN 10 mM formic acid without any extraction was used
• four water samples spiked at four different concentration levels (0.1, 0.5, 1.0 and 2.0 μg/L) to prepare the calibration curve for each analytes

All the condition applied to obtain the best chromatographic separation for all the analytes was reported in Table 1.

**Table 1:** Phase A: water 10 mM formic acid; Phase B: acetonitrile 10 mM formic acid; %As: % phase A start time; %Af: % phase A end time.

Start time → end time (min)	Column temp (°C)	Flux (µl/min)	%As→ %Af
0 → 4	40	450	90 →70
4 → 8	40	450	70 → 30
8 → 10	40	450	30 → 0
10 → 12	40	450	0 → 0
12 → 13	40	450	0 → 90
13 → 16	40	450	90 → 90

Mass Spectrometry analyses were operated in:

• sensitivity mode and positive ionization
• scan time of 0.5 s
• source temperature of 130°C and desolvation temperature of 500°C
• desolvation gas flow of 1000.0 L/Hr

The lock mass signal was used to correct automatically the analytes mass detected during the experiment.

The mass experiment was carried out acquiring

• the molecular ion with a full scan 50-1200
• the specific fragment for each toxins applying the experimental condition reported in Table 2.

**Table 2:** Experimental conditions for the selected cyanotoxins; * bicharged ion.

Toxin	Molecular ion	CE (eV)	Fragment	Retention time (min)	LOD (μg/L)
MC-RR	519.7902*	50	135.0806	5.44	0.002
[D-Asp3]-MC-RR	512.7824*	30	135.0806	5.25	0.002
MC-YR	1045.5353	60	135.0806	6.96	0.002
[D-Asp3]-MC-LR	981.5404	80	135.0806	7.22	0.020
MC-LR	995.556	80	135.0806	7.15	0.002
MC-LA	910.492	70	135.0806	8.26	0.017
MC-LY	1002.5183	60	135.0806	8.33	0.004
MC-LW	1025.5342	60	135.0806	8.63	0.022
MC-LF	986.5233	60	135.0806	8.73	0.031
MC-WR	1068.5513	70	135.0806	7.46	0.022
MC-Htyr	1059.551	60	135.0806	7.03	0.004
MC-HilR	1009.5717	100	135.0806	7.38	0.002
CYP-1041	1041.4807	60-90	150.0918	7.18	0.012
CYP-1007	1007.5197	50-90	150.0918	6.61	0.012
Microginin-690	691.3371	50-80	128.1436	4.56	0.008
Microginin-690 me	705.3528	60-90	128.1436	5.34	0.034
Microginin 527	528.2738	30-50	128.1436	4.00	0.023
Microginin 527 me	542.2894	50-90	128.1436	4.62	0.009
Microginin-704	705.3528	60-90	128.1436	5.34	0.047
Anab B	837.4618	90	201.0987	4.29	0.004
Anab A	844.424	60	84.0860	5.98	0.003
Nodularin (I.S.)	825.4505	60	135.0806	6.26

It is necessary the presence of molecular and fragment ion, at the same retention time (reported in Table 2), to be sure to detect the analites.

**Fig. 1:**
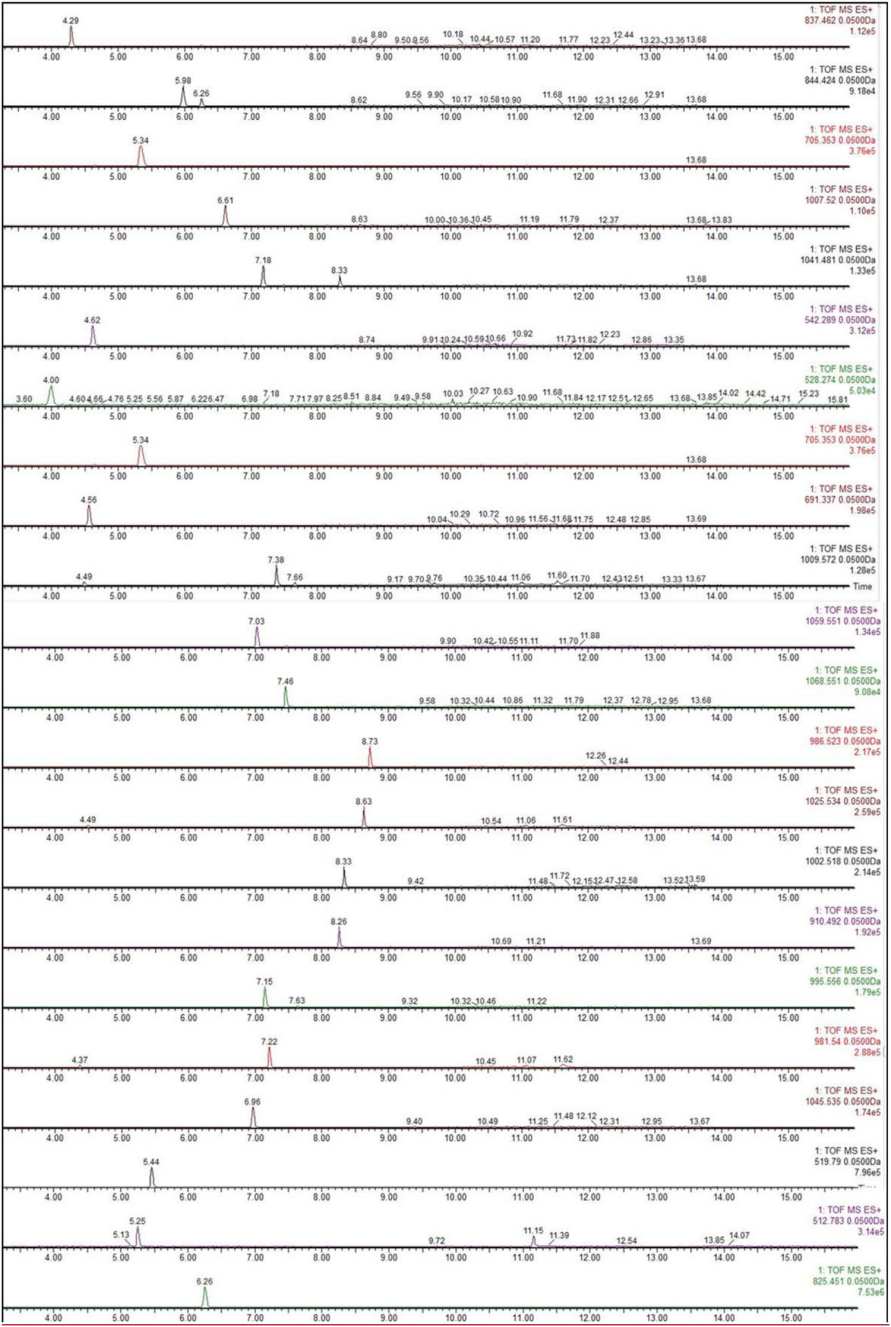
Chromatogram obtained from the analysis of a standard solution containing all analytes at 0.1 µg/L in MS mode.

The chromatograms obtained analyzing a solution containing all the selected toxins 0.1 µg/L and Nodularin 1 µg/L is reported in Fig 1 (MS mode) and 2 (MS/MS mode).

**Fig. 2:**
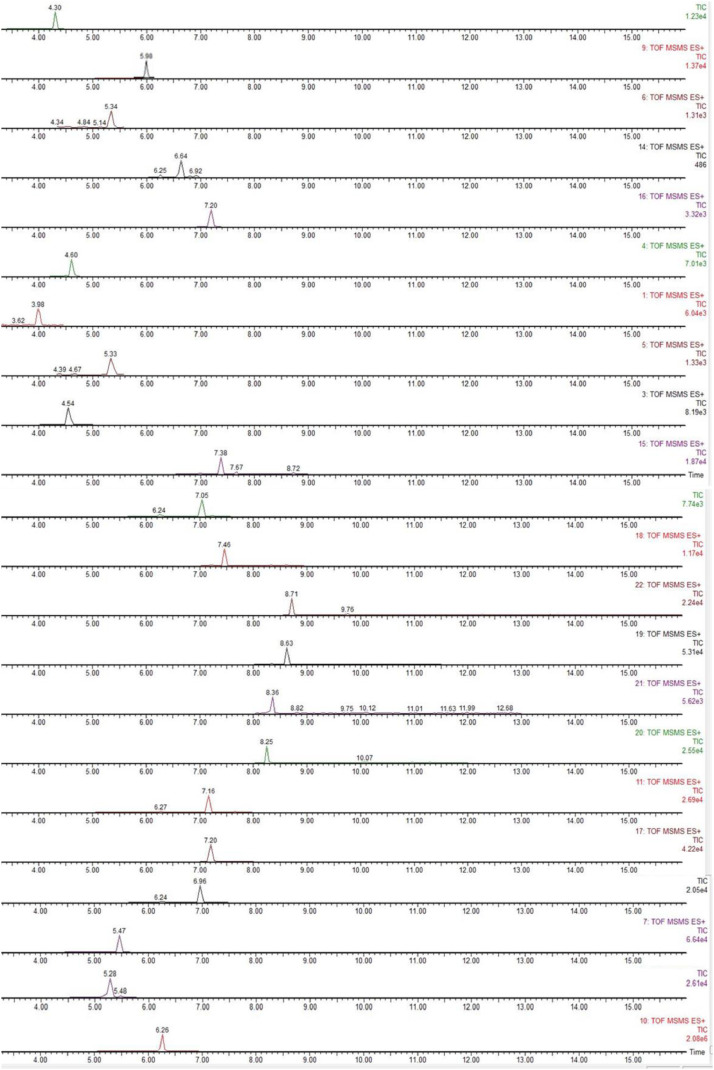
Chromatogram obtained from the analysis of a standard solution containing all analytes at 0.1 µg/L in MS/MS mode.

The method has proven to be robust, precise and accurate with recovery percentages above 85% and with relative standard deviations ≤16%, fitting for the intended purposes at the concentrations of interest.

No matrix effect ware observed for all the selected analytes.

The LODs were in the range 0.002 to 0.047 µg/L, as reported in Table 2, and all the obtained values at least 20-fold lower than the guideline value proposed by WHO for drinking water (1.0 µg/L for microcystin-LR). [2]

The limit of quantitation (LOQ) could be calculated by multiplying for 3 the LODs values.

The described method allows to detect and quantify the presence of 21 cyanotoxins of different classes in water samples for human consumption simultaneously, in short time of analysis (16 minutes) with a good resolution for all the analytes.

## Declaration of Competing Interest

The authors declare that they have no known competing financial interests or personal relationships that could have appeared to influence the work reported in this paper.

## References

[1] Nigro Di Gregorio F., Bogialli S., Ferretti E., Lucentini L. (2017). First evidence of MC-HtyR associated to a Plankthothrix rubescens blooming in an Italian lake based on a LC-MS method for routinely analysis of twelve microcystins in freshwaters. Microchem. J..

[2] World Health Organization, Guidelines for drinking-water quality: fourth edition incorporating the first addendum., 2017. http://apps.who.int/iris/bitstream/handle/10665/254637/9789241549950-eng.pdf;jsessionid=B7A8C40C69D1C876A6809D9572C6B29C?sequence=1. 28759192

